# The global cancer mental health survey: insights from patient and provider experiences on psychosocial care access

**DOI:** 10.1016/j.eclinm.2026.104047

**Published:** 2026-07-09

**Authors:** Juan P. Borda, Gilla K. Shapiro, William E. Rosa, Cristiane Bergerot, Loreto Fernández González, Wendy W.T. Lam, Anja Mehnert-Theuerkauf, M. Jean Jackson, Julia Maues, Surendran Veeraiah, Chioma Asuzu, Cara MacInnis, Madeline Li

**Affiliations:** a Department of Psychiatry, Schulich School of Medicine & Dentistry, Western University, London, ON, Canada; b Consultation-Liaison Psychiatry Service, London Health Science Center, London, ON, Canada; c Department of Supportive Care, Princess Margaret Cancer Centre, Toronto, Canada; d Department of Psychiatry, University of Toronto, Toronto, Canada; e Department of Psychiatry and Behavioral Sciences, Memorial Sloan Kettering Cancer Center, New York, USA; f Oncoclinicas & Co - Medica Scientia Innovation Research (MEDSIR), Sao Paulo, Brazil; g Cancer Research Department, Instituto Oncológico Fundación Arturo López Perez, Chile; h School of Public Health, The University of Hong Kong, Hong Kong SAR, China; i Department of Medical Psychology and Medical Sociology, University of Leipzig Medical Center, Leipzig, Germany; j Faculty of Theology, Huron University College, Western University, London, ON, Canada; k Patient Advocate, GRASP (Guiding Researchers and Advocates to Scientific Partnerships), Washington, DC, USA; l Department of Psycho-Oncology & RCTC, Cancer Institute (WIA), Chennai, India; m Department of Counselling & Human Development Studies, University of Ibadan, Nigeria; n Department of Psychology, Acadia University, Wolville, NS, Canada

**Keywords:** Psychosocial oncology, Psychosocial care, Supportive care, Cancer care, Mental health, Access to care, Health system barriers, Global health, Stigma

## Abstract

**Background:**

Psychosocial oncology (PSO) is essential to comprehensive cancer care, yet access and delivery remain inconsistent and inadequate globally. This study mapped patient- and provider-reported experiences of barriers shaping PSO access globally.

**Methods:**

Cross-sectional, web-based surveys were administered separately to adults with a self-reported cancer diagnosis and to oncology healthcare providers (HCPs) in five languages between November 12, 2024 and April 25, 2025. Descriptive statistics and multivariable logistic regressions examined associations between sociodemographic and clinical factors with PSO access, perceptions, and delivery.

**Findings:**

The final sample included 200 patients from 16 countries and 237 HCPs from 38 countries. Among patients, 85.0% rated PSO care as highly important, with 81.0% considering it as important as biomedical care, yet 63.5% reported receiving no PSO care. Among HCPs, 53.2% indicated PSO is not routinely provided at their institutions. Mental health stigma and cultural norms affecting comfort discussing emotional concerns were commonly reported barriers, reported by 57.0% and 64.0% of patients, respectively, and 52.7% and 74.3% of HCPs. Workforce and training gaps were substantial, with 66.6% of HCPs reporting insufficient specialized staff, and 42.1% reporting no formal training in PSO. Investment in PSO research was perceived as low, with 60.3% of HCPs estimating that <10% of national cancer research funding was allocated to PSO and 69.6% viewing overall research support as insufficient.

**Interpretation:**

Patients and providers emphasized the importance of PSO; however, PSO was reported to be under-delivered worldwide due to stigma, cultural factors, workforce and training limitations, and insufficient research investment. System-level strategies are needed to address these challenges and close the global PSO care gap.

**Funding:**

This study was supported by a seed grant from the Global Institute of Psychosocial, Palliative and End-of-Life Care (GIPPEC), Toronto, Canada.

**Research in context**

**Evidence before this study**

This study was conducted within the Mental Health Working Group of the *Lancet Oncology Commission on the Human Crisis of Cancer*. Existing evidence demonstrates high levels of psychological distress among people with cancer and the effectiveness of psychosocial interventions, alongside persistent unmet needs. However, prior research has largely been limited to single countries, predominantly high-income settings, and has typically examined either patient or healthcare provider perspectives in isolation. Evidence addressing system-level barriers, including stigma, cultural norms, workforce capacity, training, and research investment, remains fragmented. This study was designed to address these gaps and to inform the Commission’s recommendations aimed at redressing the imbalance between biomedical and psychosocial dimensions of cancer care and supporting expansion of PSO services worldwide.

**Added value of this study**

This study provides a global, multi-country assessment of psychosocial oncology by integrating patient- and healthcare provider-reported experiences from 16 to 38 countries, respectively. To our knowledge, no previous study has combined patient and provider perspectives across multiple global settings to examine system-level barriers such as stigma, cultural norms, workforce capacity, training, and research investment. Across settings, both groups identified a substantial gap between the perceived importance of PSO and its routine delivery in cancer care. The findings quantify shared structural and cultural barriers across settings due to stigma, workforce and training limitations, insufficient investment in PSO research, and identify factors associated with access to and delivery of care across diverse health systems.

**Implications of all the available evidence**

PSO remains insufficiently integrated into cancer care globally. Addressing this gap will require system-level strategies to embed PSO into routine oncology pathways, expand and train the workforce, reduce stigma and cultural barriers, and strengthen investment in PSO research. Future research should focus on scalable and context-sensitive models of psychosocial care, particularly in low- and middle-income countries, to advance equitable, person-centred cancer care.

## Introduction

Patients with cancer face a markedly higher risk of developing mental health disorders compared to the general population, driven by multiple factors including preventable emotional distress, treatment burden, and uncertainty about prognosis.^1^ In fact, depression, anxiety, and adjustment disorders affect nearly one-third of patients with cancer^2^ and are associated with worse clinical outcomes. Evidence shows that patients with depression have a 38% higher overall mortality risk, with cancer-specific increases ranging from 23% in breast cancer to over 80% in colorectal and prostate cancer.^3^^,^^4^ Depression and anxiety also contribute to higher recurrence risk, poorer adherence to treatment, and increased complications.^5^^,^^6^ Moreover, patients with cancer experiencing any type of emotional distress—including anxiety, depression, and general psychological distress—experience higher rates of emergency visits, hospitalizations, and outpatient care, leading to annual healthcare costs several thousand dollars greater per patient, as well as additional indirect costs such as lost income and transportation expenses.^7^^,^^8^ Beyond the impact on patients, cancer imposes substantial emotional, physical, and economic burdens on caregivers.^9^ Together, these patient- and caregiver-level consequences translate into increased demands on healthcare systems, productivity losses, and financial strain, underscoring the need for psychosocial oncology to be recognized and addressed as a core component of comprehensive cancer care and health system planning.

Nevertheless, access to effective psychosocial oncology (PSO) care remains critically insufficient worldwide,^10^ despite evidence supporting the efficacy and cost-effectiveness of psychosocial interventions.^11^ These unmet mental health needs are especially acute in low- and middle-income countries (LMICs), in patients with low socioeconomic status, and among marginalized populations, where the demand for psychosocial support is often greater but access is more limited.^10^ The *Lancet Oncology* Commission on the Human Crisis of Cancer^12^ was convened to examine the profound imbalance in cancer care, where investments disproportionately favor biomedical interventions, while the human and psychosocial dimensions often receive insufficient attention and funding. Addressing these systemic gaps is essential to reducing preventable suffering and inequities in cancer outcomes.

However, while the overall burden of mental health disorders and general emotional distress is well-documented, far fewer studies have examined the structural, cultural, and system-level factors that perpetuate these unmet needs. This study was therefore designed as a foundational global assessment to inform the Commission on the Human Crisis of Cancer by identifying the main barriers to accessing and delivering PSO services for patients with cancer, and to explore how these challenges differ across geographic, economic, and institutional settings. By examining patients’ experiences of emotional distress and unmet psychosocial needs in conjunction with healthcare providers’ perspectives on the challenges of PSO care delivery, this study aimed to generate actionable insights to inform health system planning, including resource allocation, workforce development, and integration of psychosocial services into routine oncology care pathways, to address the imbalance between biomedical and psychosocial dimensions of cancer care.

## Methods

### Study design and participants

This was a cross-sectional, web-based survey conducted in two populations: a) patients with a self-reported diagnosis of cancer and b) healthcare providers (HCPs) directly involved in oncology care or decision-making, including physicians, nurses, psychologists, social workers, researchers, and administrators/policymakers. Eligible participants were 18 years of age or older, fluent in English, Spanish, Portuguese, French, or Traditional Chinese, and had access to the internet. There were no specific exclusion criteria for this study. Formal a priori sample size calculations were not performed, as this was an exploratory, cross-sectional, web-based survey with no pre-specified primary outcome or effect size, and the target sample sizes were informed by practical considerations.

### Survey development

The survey instruments were developed by the Mental Health Working Group of the *Lancet Oncology* Commission through an iterative, collaborative process. This involved multiple rounds of review and revision among working group members via shared documentation, informed by contributions from other Commission working groups, and a narrative review of the global literature on psychosocial needs and barriers in cancer care.^10^ Two separate questionnaires were created, one for patients and one for healthcare providers, each tailored to the specific perspectives of its respondent group. The surveys specified questions about PSO care for general emotional distress provided by any healthcare provider, and mental health care provided by specialist PSO professionals.

The patient survey (see Appendix 1) consisted of 45 questions distributed across several domains: a) sociodemographic and clinical characteristics, b) access to and experience with PSO care and mental health services, c) perceived quality of care, d) stigma and cultural influences, and e) suggestions for improvement. The survey included both closed-ended and ten open-ended questions to allow deeper exploration of issues such as logistical and financial barriers, emotional and cultural challenges, and opportunities for improving PSO care. Eight of these open-ended questions were conditionally displayed based on earlier responses, while two were presented to all participants regardless of prior answers.

The healthcare provider survey (see Appendix 2) included 50 questions focused on: a) sociodemographic and professional background, b) clinical practice, c) education and training, d) health system characteristics, e) cultural influences, and f) research priorities. Multiple-choice questions consistently included an ‘other’ option with space for respondents to add complementary information.

### Pilot testing and translation

After initial development, the surveys were pre-tested with English-speaking patients (n = 5) and providers (n = 5) from five countries (Brazil, Chile, China, India, and United States). This pre-test led to minor wording adjustments to improve clarity and cultural sensitivity. A second pilot test involving ten additional participants from both respondent groups confirmed the readability and usability of the final versions.

All survey materials were subsequently translated into French, Spanish, Portuguese, and Traditional Chinese by the University Health Network Translation Services, in Toronto, Canada. These languages were chosen to maximize global reach within the limits of available resources, ensuring participation from diverse regions with diverse healthcare systems. Translations followed a forward–backward methodology to ensure conceptual and linguistic accuracy.

### Data collection

We engaged DataBrew, an international data research service company, to manage survey distribution, data collection, and analysis. Survey data were collected between November 12, 2024 and April 25, 2025 using the Typeform platform. Each population received a unique access link. Distribution followed a multi-channel strategy designed to maximize global reach and stakeholder diversity. The HCP survey was distributed through researcher and professional networks in oncology and healthcare, institutional flyers circulated among professional groups, oncology-related forums on Facebook and LinkedIn, direct outreach to cancer organisations via email and website contact forms, the ONCOLLEGE network of global oncologists, and the ecancer.com platform. The patient survey was shared through researcher networks, patient community settings, cancer survivor and support groups on Facebook and LinkedIn, and direct outreach to patient advocacy organisations. Broader dissemination was facilitated through some psychosocial oncology and oncology professional societies, leading cancer centres, and academic institutions across multiple regions. Recruitment materials emphasized inclusivity, voluntary participation, and anonymity, and included QR codes and institutional logos to enhance trust and accessibility.

Participation was voluntary and implied consent was obtained via a confirmation checkbox at the end of the study information sheet. All questions were optional except for those confirming eligibility and consent. The survey design did not include automated validation or response completeness requirements. Due to platform limitations, cookies or IP tracking were not used to prevent multiple entries. Although the likelihood of duplicate responses was low, given that no compensation was offered, a post-survey data screening process was conducted to detect potential duplicate entries by examining sociodemographic patterns and response similarities. No duplicate entries were identified. No personally identifiable information was collected.

Participants submitted their responses in the same five languages. All non-English responses for open-ended questions were translated back into English using Google Translate prior to analysis.

### Data analysis

#### Quantitative analysis

The analysis included descriptive and multivariable statistical approaches to examine barriers to accessing and delivering PSO care. We synthesized data into four major categories: (1) access to PSO care, (2) stigma and cultural factors (3), staffing and training, and (4) PSO research investment. To examine barriers to accessing PSO services and factors influencing healthcare providers’ ability to deliver PSO care, multivariable logistic regression models were constructed separately for the patient and healthcare provider datasets. Prior to building the multivariable logistic regression models, bivariable analyses were performed to identify potential associations between an individual predictor and outcome.

To assess multicollinearity, variance inflation factors (VIFs) were calculated, with all predictors demonstrating VIF values below 2, indicating no concerning levels of collinearity. Results from the regression models were presented as odds ratios (ORs) with corresponding 95% confidence intervals (CIs) and p-values, with statistical significance defined as p < 0.05. To evaluate the robustness of the models, a complete-case sensitivity analysis was performed. This analysis included only participants who had no missing data for the selected predictors and outcomes.

The results from the complete-case models were consistent with those obtained from the full sample, indicating that missing data did not meaningfully bias the findings. Item non-response was low across both surveys. In the patient survey, missing rates for primary quantitative outcome variables ranged from 0 to 4.0%, with higher rates observed only for optional open-ended items near the end of the survey (up to 25.0%), consistent with survey fatigue. In the HCP survey, missing rates for most quantitative items ranged from 0 to 7.6%. Full item-level missing data rates are reported in the Appendix. Missing data were handled using available-case analysis, whereby only participants with complete data on the relevant variables were included in each analysis. No imputation was applied given the low overall missingness rate and the alignment of missing patterns with survey skip logic and voluntary non-response, consistent with a missing-at-random assumption. The complete-case sensitivity analysis confirmed that this approach did not meaningfully bias the findings. Subgroup comparisons were also conducted across key geographic and institutional variables to identify any notable sampling imbalances.

#### Qualitative analysis

Qualitative data were drawn exclusively from the open-ended responses in the patient survey. Data analysis followed a hybrid approach.^13^ First, three computational techniques were applied to organize the dataset and highlight commonly recurring terms and co-occurring phrases: (1) word frequency analysis, which identified the most commonly used individual terms across responses; (2) trigram frequency analysis, which captured recurring three-word phrases to reveal contextually meaningful patterns; and, (3) topic modeling using Latent Dirichlet Allocation (LDA), which grouped semantically related terms into latent thematic clusters. These outputs were then used as a foundation for thematic analysis, in which conceptually related categories were grouped into broader themes. For example, terms such as “cost,” “insurance,” and “out-of-pocket” were grouped under financial barriers. To validate the consistency of findings, we drew random subsamples of responses and applied the three analytic techniques independently. This cross-validation allowed for assessment of thematic saturation and strengthened confidence in the emergent coding framework.

### Ethical considerations

This study was reviewed and approved by the Institutional Review Board at King’s College London (MRA-24/25-45346) and adhered to the ethical principles outlined in the Declaration of Helsinki. Participation was anonymous, and no incentives or compensation were provided.

### Role of the funding source

The funding source (Global Initiative for Psychosocial, Palliative and End-of-Life Care [GIPPEC], Toronto, Canada) had no role in study design; data collection, analysis, or interpretation; writing of the manuscript; or the decision to submit for publication.

## Results

### Participant characteristics

A total of 200 patients and 237 HCPs completed the survey. Engagement metrics, including views, starts, and submissions, are summarized in Appendix 3. Table 1 presents the sociodemographic and clinical characteristics of patients and HCPs. Patients were recruited from 16 countries and HCPs were from 38 countries, with representation from all six World Health Organization (WHO) regions in both samples. The patient sample was predominantly female and highly educated, with most participants married or partnered and insured. Clinically, most were in remission, followed by those undergoing active treatment, with a smaller proportion receiving palliative care. The healthcare provider sample was predominantly female and multidisciplinary, comprising physicians, psychologists, nurses, social workers, and smaller proportions of researchers and policymakers. Most worked in public healthcare settings, with representation from private, academic, and non-governmental sectors.

**Table 1:** Sociodemographic, clinical, and professional characteristics of participants.

	Patients n (%)	HCPs n (%)
Median age (years)	55	44
Gender (Female)	155 (77.5%)	175 (73.8%)
Higher education/Additional professional training	165 (82.5%)	
Married/Partnered	117 (58.5%)	
Private health insurance	112 (56.0%)	
Employed	80 (40.0%)	
On disability	20 (10.0%)	
Ethnicity		
White	112 (56%)	78 (32.9%)
Asian	40 (20%)	57 (24.1%)
Hispanic/Latino	16 (8%)	39 (16.5%)
Black	10 (5%)	34 (14.3%)
Middle Easter or North African	2 (1%)	20 (8.4%)
Native American or Indigenous	1 (0.5%)	1 (0.4%)
Other	19 (9.5%)	8 (3.3%)
LMIC	51 (25.5%)	112 (47.3%)
WHO region		
Americas	130 (65%)	90 (38%)
Western Pacific	37 (18.5%)	33 (13.9%)
Africa	7 (3.5%)	31 (13.1%)
Europe	3 (1.5%)	27 (11.4%)
Eastern Mediterranean	2 (1%)	24 (10.1%)
South-East Asia	3 (1.5%)	11 (4.6%)
Treatment phase		
In remission	117 (58.5%)	
Active treatment	64 (32.0%)	
Palliative care	6.0% (n = 12)	
Professional role		
Physician		81 (34.2%)
Psychologist		42 (17.7%)
Nurse		30 (12.7%)
Social work		17 (7.2%)
Researcher		9 (3.8%)
Administrator/Policy maker		5 (2.1%)
Practice setting		
Public health sector		145 (61.2%)
Private health sector		52 (21.9%)
Academic institution		22 (9.3%)
Non-governmental organization		10 (4.2%)

### Access to PSO care

Survey responses from patients and HCPs are summarized in Table 2. Among patients, 85.0% (n = 170) rated PSO as “very important,” and 81.0% (n = 162) believed it should be considered as important as biomedical treatment, yet 63.5% (n = 127) reported receiving no psychosocial support. Of those, 57.5% (n = 73) indicated they would have wanted such care, and 56.5% (n = 113) felt PSO was not sufficiently prioritized in their institutions. Among HCPs, 85.2% (n = 202) agreed PSO is equally important as biomedical care, but 53.2% (n = 126) said services were not routinely provided, 50.6% (n = 120) estimated that fewer than one-quarter of their patients received adequate care, and 48.5% (n = 115) felt PSO was undervalued where they worked.

**Table 2:** Patient and healthcare provider responses to close-ended survey items.

Question	Response	HCPs n (%)	Patients n (%)
Access to PSO care			
Is mental health care routinely provided to all patients in your institution?	Yes	104 (43.9%)	
Have you sought mental health support related to your cancer?	Yes		105 (52.5%)
Have you received psychosocial care from your cancer team?	Yes		72 (36%)
Have you wanted to receive psychosocial care?	Yes		73 (57.5%)
How satisfied are you with the psychosocial care from your cancer team such as your oncologist or nurse?	Very unsatisfied		1 (0.5%)
	Somewhat unsatisfied		6 (3%)
	Neutral		8 (4%)
	Somewhat satisfied		27 (13.5%)
	Very satisfied		30 (15%)
	NA		128 (64%)
Have you experienced any financial barriers to accessing such care?	Yes		88 (44.2%)
What proportion of your patients with cancer require mental health care?	More than 75%	66 (27.8%)	
	51–75%	59 (24.9%)	
	25–50%	73 (30.8%)	
	Less than 25%	36 (15.2)%	
	NA	3 (1.3%)	
In your opinion what proportion of your patients who have or had cancer are receiving sufficient mental health care?	More than 75%	13 (5.5%)	
	51–75%	22 (9.3%)	
	25–50%	77 (32.5%)	
	Less than 25%	120 (50.6%)	
	NA	5 (2.1%)	
What proportion of family caregivers of your patients with cancer require mental health care?	More than 75%	49 (20.7%)	
	51–75%	44 (18.6%)	
	25–50%	78 (32.9%)	
	Less than 25%	55 (23.2%)	
	NA	11 (4.6%)	
How do you perceive the importance of psychosocial oncology care relative to biomedical oncology care?	Psychosocial care is more important than medical care	16 (6.8%)	10 (5%)
	Psychosocial care is equally important as medical care	202 (85.2%)	162 (81%)
	Psychosocial care is less important than medical care	11 (4.6%)	20 (10%)
	NA	8 (3.4%)	8 (4%)
Do you think there is sufficient prioritization for psychosocial oncology care in your institution?	Yes	60 (25.3%)	
Do you feel that mental health care is given enough importance in your care setting?	Yes		39 (19.5%)
Do you feel that psychosocial oncological care is given enough importance in your care setting?	Yes		46 (23%)
How important do you think psychosocial care is in cancer?	Very unimportant		3 (1.5%)
	Somewhat unimportant		1 (0.5%)
	Neutral/Not sure		9 (4.5%)
	Somewhat important		17 (8.5%)
	Very important		170 (85%)
Stigma and cultural factors			
How prevalent is the stigma related to emotional struggles and mental health care among patients with cancer in your country?	Very rare	6 (2.5%)	2 (1%)
	Rare	18 (7.6%)	22 (11.1%)
	Moderate	87 (36.7%)	56 (28%)
	Common	73 (30.8%)	60 (30%)
	Very common	52 (21.9%)	54 (27%)
	NA	1 (0.4%)	6 (3%)
Have you ever felt stigma or judgement when seeking psychosocial or mental health care?	Yes		68 (34%)
Do you feel comfortable discussing your psychosocial or mental health needs with your oncology care team?	Yes		140 (70%)
In your opinion can anything be done to reduce stigma around seeking psychosocial and mental health care?	Yes		179 (89.5%)
To what extent do cultural values in your country influence healthcare providers willingness to engage in conversations about emotional or mental health issues with their patients?	Do not influence at all	4 (1.7%)	7 (3.5%)
	Rarely influence	17 (7.2%)	13 (6.5%)
	Neutral/No influence	37 (15.6%)	44 (22%)
	Somewhat influence	109 (46%)	74 (37%)
	Strongly influence	67 (28.3%)	54 (27%)
	NA	3 (1.3%)	8 (4%)
Staffing and training			
How sufficient is mental health care staffing at your institution?	Very insufficient	29 (12.2%)	
	Insufficient	129 (54.4%)	
	Neutral	37 (15.6%)	
	Sufficient	34 (14.3%)	
	Very sufficient	7 (3%)	
How would you rate the availability of mental health care services in your area?	Very poor		13 (6.5%)
	Poor		38 (19%)
	Fair		71 (35.5%)
	Good		44 (22%)
	Very good		25 (12.5%)
	NA		9 (4.5%)
What mental health care disciplines do you have available at your institution?	Psychology	169 (71.3%)	
	Social work	156 (65.8%)	
	Psychiatry	142 (59.9%)	
	Chaplain/Clergy	90 (37.9%)	
	Lay/Peer support	77 (32.5%)	
	Mental health nursing	63 (28.6%)	
	Other	11 (4.6%)	
How comfortable are healthcare professionals in your institution in providing psychosocial care?	Very uncomfortable	9 (3.8%)	
	Somewhat uncomfortable	33 (13.9%)	
	Neutral	59 (24.9%)	
	Somewhat comfortable	91 (38.4%)	
	Very comfortable	44 (18.6%)	
	NA	1 (0.4%)	
Have you received any training in psychosocial oncology?	No, not interested	6 (2.5%)	
	No, but interested	75 (31.6%)	
	Not aware of such training	19 (8%)	
	Yes	119 (50.2%)	
	NA	18 (7.6%)	
How would you rate the accessibility of training opportunities in psychosocial oncology for healthcare professionals in your country?	Very inaccessible	32 (13.5%)	
	Somewhat inaccessible	76 (32.1%)	
	Neutral	39 (16.5%)	
	Somewhat accessible	71 (30%)	
	Very accessible	17 (7.2%)	
	NA	2 (0.8%)	
How motivated are healthcare professionals in your institution to seek training in psychosocial oncology?	Vey unmotivated	12 (5.1%)	
	Somewhat unmotivated	40 (16.9%)	
	Neutral	71 (30%)	
	Somewhat motivated	71 (30%)	
	Very motivated	43 (18.1%)	
What is the main factor influencing whether psychosocial oncology training is prioritized at your institution?	Institutional policies	67 (28.3%)	
	Costs	74 (31.2%)	
	Personal interest	39 (16.5%)	
	Lack of time	39 (16.5%)	
	NA	12 (5.1%)	
	Patient advocacy	4 (1.7%)	
	Other	2 (0.8%)	
What are the 3 main factors limiting healthcare professionals’ access to such training opportunities in your country?	Workload and time constraints	134 (56.5%)	
	Financial cost	121 (46%)	
	Institutional support	114 (44.7%)	
	Unavailability of such training	104 (43.8%)	
	Lack of awareness	97 (40.9%)	
	Lack of interest	45 (18.9%)	
	Geographical location	28 (11.8%)	
	Language	4 (1.7%)	
	There are no factors limiting access	2 (0.8%)	
	Other	2 (0.8%)	
PSO research investment			
What proportion of all oncology research funding do you think is allocated to psychosocial oncology care in your country?	<1%	84 (35.4%)	
	1–10%	59 (24.9%)	
	10–20%	15 (6.3%)	
	>20%	6 (2.5%)	
	Don’t know	73 (30.8%)	
Is there sufficient funding for psychosocial oncology research in your country?	Very insufficient	69 (29.1%)	
	Insufficient	96 (40.5%)	
	Neutral	44 (18.6%)	
	Sufficient	4 (1.7%)	
	Very sufficient	2 (0.8%)	
	NA	22 (9.3%)	
Do you assess psychosocial outcomes in your research?	No, but I plan to in the future	55 (23.2%)	
	No, psychosocial outcomes are not relevant in my research	20 (8.4%)	
	I don’t conduct research	79 (33.3%)	
	Yes	79 (33.3%)	
	NA	4 (1.7%)	
What do you think are the top 3 research priorities in psychosocial oncology?	Impacts of psychosocial care on cancer outcomes	154 (65%)	
	Understanding the causes of psychosocial distress in patients with cancer	137 (57.8%)	
	Survivorship	103 (43.5%)	
	End-of-life care and bereavement	101 (42.6%)	
	Novel psychosocial interventions	84 (35.4%)	
	Psychosocial strategies in the prevention and detection of cancer	68 (28.7%)	
	Cross-cultural studies	34 (14.3%)	
	Other	3 (1.3%)	

### Stigma and cultural factors

**Fig. 1:**
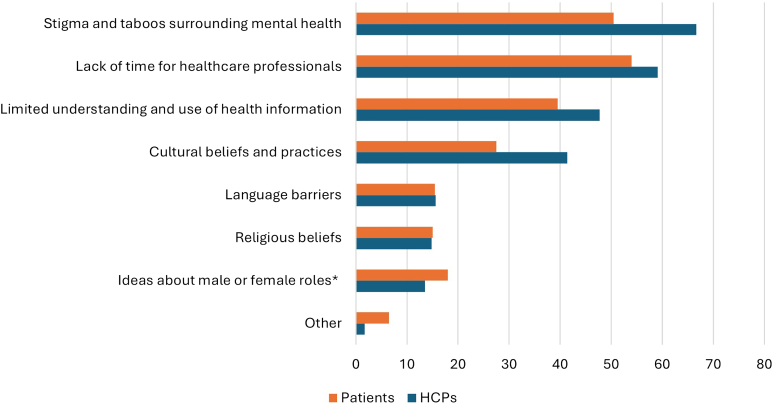
**Barriers to communication about mental health reported by patients and healthcare providers.** Note: ∗ Ideas about male or female roles” refers to gender norms influencing comfort in expressing emotional distress or seeking psychological support (e.g., expectations to appear strong, avoid vulnerability, or not burden others), which may limit discussion of mental health concerns during clinical encounters.

More than half of patients (57.0%, n = 114) reported that stigma surrounding mental health was common, 34.0% (n = 68) had personally experienced stigma, and 28.0% (n = 56) felt uncomfortable discussing psychosocial concerns with their oncology team. Cultural values were reported to shape comfort in seeking psychosocial support and discussing mental health needs in 64.0% (n = 128) of patients. Among HCPs, 52.7% (n = 125) acknowledged stigma in their practice environments, and 74.3% (n = 176) reported cultural values influenced their willingness to engage in mental health conversations. Fig. 1 summarizes the most frequently endorsed barriers to communication about mental health issues.

### Staffing and training

Staffing and training gaps in PSO and mental health care were widely recognized. Among patients, 35.5% (n = 71) rated PSO availability as “fair” and 25.5% (n = 51) as “poor.” Two-thirds of HCPs (66.6%, n = 158) reported insufficient specialized staff. Psychologists (71.3%, n = 169), social workers (65.8%, n = 156), and psychiatrists (59.9%, n = 142) were most commonly available, while fewer institutions reported access to chaplains (37.9%, n = 90), peer support workers (32.5%, n = 77), or mental health nurses (26.6%, n = 63). Training was inconsistent: 42.1% (n = 100) reported no formal preparation, and many cited barriers such as time constraints (56.5%, n = 134), financial costs (51.1%, n = 121), lack of institutional support (48.1%, n = 114), and limited availability (43.9%, n = 104).

### PSO research investment

Most HCPs (60.3%, n = 143) estimated PSO received less than 10% of national cancer research funding, and 69.6% (n = 165) judged overall support insufficient. Only 36.0% (n = 85) reported that institutional or pharmaceutical incentives in their settings supported mental health research. While 31.6% (n = 75) did not currently address psychosocial outcomes, 73.3% (n = 55) of this group expressed interest in doing so. Priority research areas included the impact of PSO on cancer outcomes (65%, n = 154), causes of distress (57.8%, n = 137), survivorship (43.5%, n = 103), and end-of-life care (42.6%, n = 101).

### Multivariable analysis—patients

Table 3 presents the multivariable analysis of predictors identified in the bivariable analysis. Several sociodemographic and clinical factors were significantly associated with patients’ engagement in psychosocial care and their perceptions of its importance and accessibility. Older patients were significantly less likely to have sought mental health care (OR 0.94, 95% CI 0.92–0.97, p = 0.001), to have received PSO support from their oncology team (OR 0.96, 95% CI 0.93–0.98, p = 0.002), and to report financial barriers to accessing PSO care (OR 0.96, 95% CI 0.93–0.99). Male patients were less likely to have sought mental health care (OR 0.18, 95% CI 0.06–0.47, p = 0.001), to express a desire for psychosocial support (OR 0.34, 95% CI 0.12–0.93, p = 0.035), and to report experiencing stigma when accessing such services (OR 0.37, 95% CI 0.13–0.94, p = 0.045). Consistently, men were more likely to perceive adequate prioritization of PSO care (OR 2.67, 95% CI 1.15–6.47, p = 0.025).

**Table 3:** Multivariable associations between sociodemographic/clinical predictors and patient responses.

	Perceived adequate prioritisation of PSO care	Perceived mental health care as important	Wanted PSO care	Sought mental health support	Received PSO care from cancer team	Reported financial barriers to PSO care	Comfortable discussing mental health needs with oncology team	Perceived mental health stigma as common	Personally experienced mental health stigma when seeking care	Perceived influence of cultural values on HCP willingness to discuss mental health	Belief that mental health stigma can be reduced
	OR (95% CI)	OR (95% CI)	OR (95% CI)	OR (95% CI)	OR (95% CI)	OR (95% CI)	OR (95% CI)	OR (95% CI)	OR (95% CI)	OR (95% CI)	OR (95% CI)
Age	1.01 (0.98–1.04)	1.00 (0.96–1.03)	0.96 (0.92–1.00)	0.94^b^ (0.91–0.97)	0.96^a^ (0.93–0.98)	0.96^a^ (0.93–0.99)	0.98 (0.95–1.01)	1.00 (0.98–1.03)	0.99 (0.96–1.02)	1.00 (0.98–1.03)	1.02 (0.97–1.07)
Male Gender	2.67^a^ (1.15–6.47)	1.12 (0.45–2.71)	0.34^a^ (0.12–0.92)	0.18^b^ (0.06–0.47)	0.69 (0.27–1.67)	0.64 (0.24–1.65)	1.22 (0.47–3.41)	1.52 (0.66–3.69)	0.37 (0.13–0.94)^a^	1.97 (0.78–5.51)	0.45 (0.13–1.63)
Ethnic background	1.91 (0.79–4.70)	1.32 (0.50–3.41)	1.23 (0.38–4.14)	0.68 (0.25–1.843)	0.52 (0.20–1.29)	1.79 (0.72–4.48)	1.40 (0.51–4.10)	2.09 (0.90–5.09)	0.96 (0.39–2.36)	1.60 (0.66–4.00)	1.67 (0.34–10.04)
LMIC	1.52 (0.58–4.08)	1.72 (0.62–4.93)	0.81 (0.23–2.75)	0.18^a^ (0.05–0.54)	1.41 (0.5–3.98)	0.97 (0.34–2.69)	0.96 (0.32–3.03)	1.42 (0.5–4.17)	1.03 (0.37–2.85)	1.54 (0.53–4.75)	1.16 (0.19–7.38)
Has health insurance	1.06 (0.48–2.36)	1.01 (0.45–2.29)	1.08 (0.40–2.94)	0.86 (0.34–2.11)	1.32 (0.60–2.95)	0.41^a^ (0.18–0.89)	3.31^a^ (1.40–8.15)	0.65 (0.30–1.38)	0.57 (0.26–1.24)	0.81 (0.35–1.81)	0.89 (0.20–3.68)
Time since diagnosis >5 years	1.44 (0.66–3.19)	0.70 (0.31–1.54)	NA	1.39 (0.61–3.28)	1.34 (0.61–2.99)	1.41 (0.63–3.23)	1.58 (0.67–3.75)	0.96 (0.46–1.98)	1.43 (0.65–3.20)	0.97 (0.44–2.11)	0.12^a^ (0.02–0.57)
Palliative treatment phase	0.61 (0.12–2.85)	0.44 (0.08–2.20)	0.55 (0.08–4.15)	0.42 (0.08–2.48)	1.30 (0.27–5.92)	0.34 (0.04–1.82)	0.45 (0.09–2.45)	0.43 (0.08–2.26)	0.42 (0.06–2.11)	0.24 (0.05–1.14)	0.56 (0.05–13.26)
*Remission phase*	0.40^a^ (0.16–0.92)	0.79 (0.33–1.86)	1.20 (0.41–3.51)	0.622 (0.22–1.67)	1.01 (0.43–2.41)	1.45 (0.61–3.58)	1.20 (0.46–3.08)	2.04 (0.9–4.71)	1.27 (0.53–3.09)	0.81 (0.32–1.99)	3.44 (0.77–16.52)
Self-employed/Part-time employed	0.35 (0.10–1.13)	0.57 (0.14–1.98)	0.88 (0.24–3.29)	1.75 (0.51–6.18)	0.66 (0.18–2.12)	1.63 (0.54–4.96)	0.42 (0.13–1.30)	0.71 (0.25–2.02)	0.99 (0.30–3.05)	0.57 (0.20–1.68)	0.60 (0.11–3.65)
Not working/On Disability	0.87 (0.39–1.93)	2.98^a^ (1.28–7.27)	1.82 (0.60–5.79)	3.47^a^ (1.39–9.21)	2.02 (0.9–4.7)	1.46 (0.63–3.44)	1.87 (0.72–4.97)	1.60 (0.74–3.50)	1.66 (0.73–3.84)	1.58 (0.68–3.69)	1.81 (0.45–7.29)

Notes: PSO = psychosocial oncology; HCP = healthcare provider; LMIC = low- and middle-income country; OR = Odds ratio; CI = Confidence interval. Multivariable models adjusted for other listed covariates. Reference categories: female gender, white ethnicity, high-income country, no health insurance, time to diagnosis ≤5 year, active treatment phase, and full-time employment.

a p < 0.05.

b p < 0.01.

Patients in LMICs were significantly less likely to have accessed mental health services (OR 0.18, 95% CI 0.053–0.539, p = 0.003). Being unemployed or on disability was associated with higher odds of having sought mental health care (OR 3.47, 95% CI 1.39–9.21, p = 0.010), and these individuals were also more likely to rate psychosocial care as very important (OR 2.98, 95% CI 1.28–7.27, p = 0.013). Insurance coverage further shaped perceptions, with privately insurance patients more likely to perceive psychosocial care as very important (OR 3.26, 95% CI 1.10–10.44, p = 0.037), and to report comfort in discussing their mental health with their oncology team (OR 3.31, 95% CI 1.40–8.15, p = 0.007). Patients in remission were less likely to perceive that psychosocial care had been prioritized in their treatment (OR 0.40, 95% CI 0.16–0.92, p = 0.035). Those more than five years post-diagnosis were less likely to believe that stigma around mental health and PSO care could be addressed and reduced (OR 0.12, 95% CI 0.02–0.57, p = 0.010).

### Multivariable analysis—healthcare providers

**Fig. 2:**
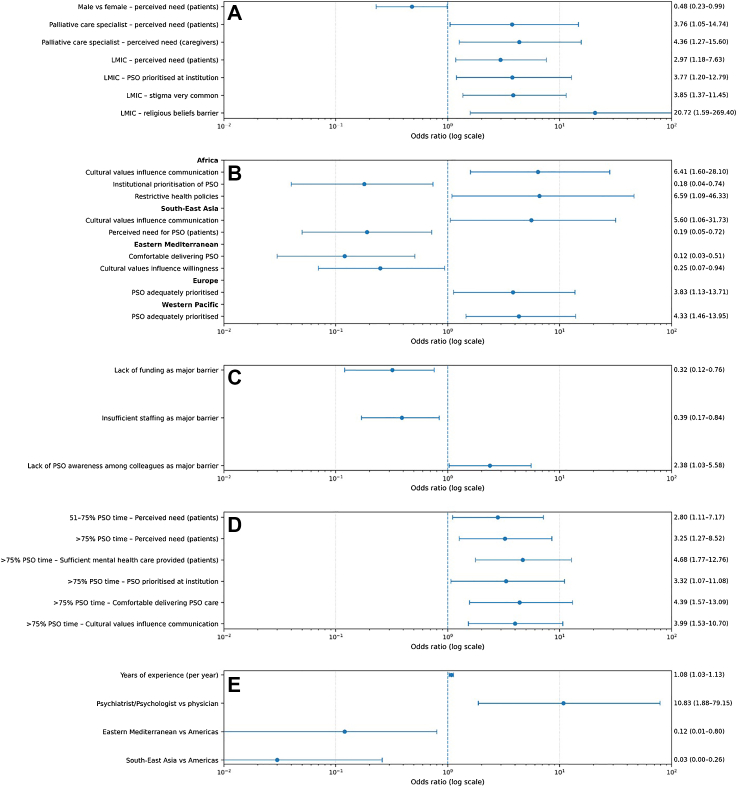
**Multivariable significant predictors of psychosocial oncology perceptions, barriers, and training among healthcare providers.** Note: Forest plots display adjusted Ors with 95% Cis from multivariable logistic regression models examining provider-level, institutional, and regional factors associated with perceptions and delivery of PSO care. **Panel A** shows sociodemographic and professional background predictors of perceived need for psychosocial care, institutional prioritisation, stigma, and belief-related barriers. **Panel B** shows regional predictors of perceptions related to PSO need, prioritisation, comfort delivering care, and cultural or policy-related barriers, stratified by WHO region. **Panel C** presents institutional and practice-level predictors of perceived structural barriers to PSO care, including resource constraints and professional awareness. **Panel D** illustrates associations between degree of professional involvement in PSO (proportion of clinical time dedicated to PSO) and perceptions of need, adequacy, prioritisation of care, and provider comfort. **Panel E** depicts predictors of having received formal PSO training, including years of professional experience, primary professional role, and WHO region. All models were adjusted for relevant covariates as specified in Supplementary Table S4.

The multivariable analysis of provider characteristics and perceptions of PSO needs, delivery, and barriers is summarised in Fig. 2, presenting statistically significant predictors only. The complete regression results provided in Appendix 4 (Supplementary Table S4). Sociodemographic and professional background characteristics were associated with multiple outcomes (Fig. 2A): male providers were less likely to perceive patient mental health needs (OR 0.48, 95% CI 0.23–0.99, p < 0.05), whereas palliative care specialists more frequently recognised psychosocial support needs among both patients (OR 3.76, 95% CI 1.05–14.74, p < 0.05) and caregivers (OR 4.36, 95% CI 1.27–15.60, p < 0.05). Each additional year of professional experience was associated with a 7.5% increase in the likelihood of having received PSO training (OR 1.08, 95% CI 1.03–1.13, p < 0.001), and with greater comfort in providing psychosocial care (OR 1.05, 95% CI 1.01–1.09, p < 0.05). Providers working in low- and middle-income countries more often reported unmet mental health needs among patients (OR 2.97, 95% CI 1.18–7.63, p < 0.05), greater institutional prioritisation of psychosocial care (OR 3.77, 95% CI 1.20–12.79, p < 0.05), and more frequently perceived stigma and belief-based barriers to communication about mental health (OR 3.85, 95% CI 1.37–11.45, p < 0.05).

Patterns also differed by geographic region (Fig. 2B). Providers from South-East Asia (OR 0.19, 95% CI 0.05–0.72, p < 0.05) and Africa (OR 0.18, 95% CI 0.04–0.74, p < 0.05) were less likely to perceive psychosocial care as adequately prioritised or needed, yet more likely to identify cultural norms, restrictive policies, and belief-based barriers as obstacles to care delivery. In contrast, respondents from Europe (OR 3.83, 95% CI 1.13–13.71, p < 0.05) and the Western Pacific region (OR 4.33, 95% CI 1.46–13.95, p < 0.05) were more likely to report institutional prioritisation of PSO care. Providers from the Eastern Mediterranean region reported lower comfort in delivering psychosocial care (OR 0.12, 95% CI 0.03–0.51, p < 0.05), were less likely to perceive cultural values as influencing provider–patient communication (OR 0.25, 95% CI 0.07–0.94, p < 0.05).

Institutional context influenced perceived barriers (Fig. 2C) with psychiatrists and psychologists significantly more likely to have received PSO training compared to physicians (OR 10.83, 95% CI 1.88–79.15, p < 0.05). Greater professional engagement in psychosocial oncology was associated with more favourable perceptions of need, delivery, and prioritisation of care (Fig. 2D): providers dedicating 51–75% of clinical time to PSO were more likely to perceive patient mental health needs (OR 2.80, 95% CI 1.11–7.17, p < 0.05) and to report cultural values as influencing communication (OR 4.45, 95% CI 1.72–11.80, p < 0.05), while those dedicating more than 75% of clinical time to PSO were more likely to perceive patient mental health needs (OR 3.25, 95% CI 1.27–8.52, p < 0.05), report sufficient PSO delivery (OR 4.68, 95% CI 1.77–12.76, p < 0.05), perceive adequate institutional prioritisation (OR 3.32, 95% CI 1.07–11.08, p < 0.05), and report cultural values as influencing communication (OR 3.99, 95% CI 1.53–10.70, p < 0.05). Providers dedicating 25–50% of clinical time to PSO were more likely to have received PSO training (OR 2.81, 95% CI 1.01–8.24, p < 0.05). Receipt of psychosocial oncology training varied by professional experience, clinical role, and WHO region, with substantial regional disparities in training exposure; each additional year of professional experience was associated with a 7.5% increase in the likelihood of having received PSO training (Fig. 2E).

### Qualitative analysis

Open-ended responses from patients provided valuable context to complement the quantitative findings, offering insight into the lived experience of accessing PSO care. Thematic saturation was reached between 75 and 80% of the sample for most questions. Table 4 presents the ten open-ended questions along with the corresponding themes and illustrative quotes. The analysis revealed six overarching themes that framed patient experiences: barriers to accessing psychosocial support; helpful aspects of care received; gaps in care and suggestions for improvement; stigma and cultural influences; communication with oncology teams; and proposed strategies to reduce stigma and improve access.

**Table 4:** Open-ended survey questions, emergent themes, and illustrative quotes.

Question	Themes	Illustrative Quote
1. Could you describe the specific challenges you have experienced? (e.g., scheduling difficulties, availability of services at different locations, transportation issues, etc.)	Availability and cost-related challenges	40-year-old Latin male from South America with colorectal cancer: “The cost of psychological counselling is too high”
	Long wait times	40-year-old white female from North America with breast cancer: “I was treated at two different cancer centres. At both, there was a long wait”
	Finding practitioners with cancer-specific experience	54-year-old Eastern Mediterranean female from North of Africa with leukemia: “There is a shortage of professionals trained in psycho-oncology.”
2. Please describe any financial barriers you have experienced to accessing mental health care.	Accessing psychosocial care is not affordable (especially for unemployed, on disability, on leave from work, retired)	50-year-old Asian female from Western Pacific with breast cancer: “Treatment usually requires a prolonged period of time, and the out-of-pocket expenses represent a cost beyond what patients can typically afford in their daily lives.”
	Not covered by insurance, high out-of-pocket expenses	40-year-old white female from North America with breast cancer: “I am given one session per month with CancerCare. Anything outside of that, I need to pay for with private insurance and deductibles.”
3. What parts of the psychosocial care you received were the most helpful?	Supportive social workers and oncologists	55-year-old Latin female from South America with breast cancer: “My oncologist encourages me to talk about the issue and see mental health professionals.”
	Talking and discussing (in general)Sub-theme: talk therapy, cognitive therapy	47-year-old black female from North America with Hodgkins Lymphoma: “Just having someone to talk to who is not judgemental. Some to support me and let me know the feelings I had are normal.”
4. What parts of the psychosocial care you received could have been improved?	Barriers to access to care and support	48-year-old Asian female from Western Pacific with leukemia: “There are long waits and difficulties scheduling appointments.”
	Follow-up, ongoing care	52-year-old white female from North America with breast cancer: “Having more mental health care after treatment was over. My mental health issues developed post-treatment and up to 5 years afterwards.”
5. What psychosocial care would you have liked to receive?	Dealing with anxiety and fear	75-year-old Latin male from South America with bladder cancer: “I would like to receive support for sadness, hopelessness and doubts.”
	Support groups, counselling, community support	21-year-old Latino male from South America with Anaplastic Large Cell Lymphoma Peer support, speaking to someone that had been through something similar.”
	Sub-theme: Dealing with family interactions, communicating with family	58-year-old black female from Africa with breast cancer: “Teach our family members about cancer, so that they understand what we are going through.”
6. Please describe if you have ever felt stigma or judgement when seeking psychosocial or mental health care	Cultural discourses about cancer	59-year-old black female from Africa with sarcoma: “People expect you to be ‘strong’ all the time, as if having a bad day — or even a few bad days — is somehow wrong.”
	Judgement from family or cultural community	66-year-old Latin female from South America with melanoma: “It is part of cultural norms and popular ignorance to judge individuals who seek psychosocial or mental health support as being ‘crazy.’”
	Other judgement words—failed, crazy, exaggerated, shame	59-year-old white female from North America with lung cancer: “Admitting that I needed help made me feel that I had failed to manage myself.”
7. Please explain your comfort level of discussing your psychosocial or mental health needs with your oncology care team.	Gradually becoming more comfortable	50-year-old Asian male from North America with lung cancer: “At first I wasn’t comfortable, but now I have a better understanding of the harm of not dealing with mental health causes. I’m now much more comfortable talking to health care professionals and asking for help.”
8. Please explain what can be done to reduce stigma around seeking psychosocial and mental health care.	Make psychosocial and mental health care part of the treatment process	58-year-old white female from North America with sarcoma: “Psychosocial care should be part of the care plan for cancer patients from the start.”
	Increase awareness, education, campaigns	28-year-old Asian female from Wester Pacific with T cell Lymphoma: “Change the public opinion that seeking psychosocial oncology care is equivalent to mental illness”
	Training for clinicians, doctors, and healthcare workers around mental health issues	58-year-old black female from Africa with thyroid cancer: “Healthcare professionals need training on psychosocial oncology and how cancer affects us.”
	Doctor-led discussions about mental health issues	53-year-old white female from North America with vaginal cancer: “Patients are afraid to start the conversations. Doctors must create an opening in conversations
	Open communication, talking about it	52-year-old white female from North America with endometrial cancer: “Continue to talk about it, share stories, and let people know it is normal and give them resources to help them. Feeling not alone is key.”
9. What improvements would you suggest to make psychosocial and mental health care more accessible and acceptable for patients like you?	Make it more financially affordable (or free)	58-year-old Asian female from Western Pacific with Hodgkin Lymphoma: “More government support is needed so patients don’t have to worry about covering all the expenses of treatment.”
	Resources and information provided at hospitals, clinics	52-year-old white female from North America with breast cancer: “It would be great to have more social workers on site at the hospital as well as an AI cancer coach who can provide support and answer questions between sessions.”
	Increased availability and access to psychosocial care, professionals, and services	37-year-old Asian female from North America with T cell Lymphoma: “I suggest improving early access to mental health support, especially during diagnosis and treatment. Healthcare providers should proactively screen for mental health issues and offer referrals to counselling and support services”
	Include psychosocial care as part of the treatment	62-year-old white male from Europe with prostate cancer: “Psychosocial care should be put alongside medical treatment.”
	Support groups, peer support	36-year-old white female from Nort America with ymphoma“The team should include access to a peer mentor/navigator who has gone through the lived experience of cancer. Patients will likely feel more comfortable speaking to someone who is similar in age and experienced cancer like them. The peer mentor/navigator can assist by providing them resources, listening, and offering support so they feel heard.”
10. Are there any additional services or resources you believe should be offered to support psychosocial and mental health care for patients with cancer?	Peer, Group Support	59-year-old white female from North America with lung cancer “Peer support can be very helpful for those intimidated by the concept of psychologists or social workers.”
	Information and Education	56-year-old black female from Africa with breast cancer: “There is a need for more awareness and education programs.”
	Different types of therapy, counselling	43-year-old Latino female from South America with breast cancer: “Complementary therapies such as art therapy, yoga, mindfulness, expressive dance, theater, makeup workshops, decoration, self-care.”
	Free, affordable, low cost options/financial support	57-year-old Asian male from Western Pacific with leukemia: “Need for government funding to support patient groups, transportations, income, childcare etc.”

#### Barriers to accessing psychosocial support

Patients described a range of obstacles to accessing mental health care, with key themes including limited availability of services, long wait times, lack of professionals with cancer-specific expertise, and logistical challenges such as scheduling and transportation. Financial concerns were frequently reported, even among patients with insurance coverage, due to limited reimbursement or high out-of-pocket costs (see Table 4, Questions 1 and 2).

#### Helpful aspects of care received

Among patients who accessed psychosocial services, several positive elements were identified. Support from social workers and oncologists was commonly appreciated, especially when it facilitated access to resources or validated emotional needs. Therapies, including cognitive-behavioral interventions and emotional support from nonjudgmental professionals, were highlighted as particularly beneficial (Table 4, Question 3).

#### Gaps in Care and desired improvements

Patients noted delays in receiving care and a lack of continuity, particularly in the post-treatment or survivorship phase. Many expressed a desire for earlier access and sustained mental health support, especially for managing anxiety, fear of recurrence, and adjustment challenges after treatment. Suggestions included integrating PSO care earlier in the cancer trajectory, expanding peer support, and offering services to help families understand and support the patient (Table 4, Questions 4 and 5).

#### Stigma and cultural barriers

Stigma was a recurrent theme, particularly among patients who felt pressure to remain resilient or who perceived judgement from family members or cultural communities. Some participants reported internalized stigma, associating help-seeking with weakness or failure. Others described external stigma, such as feeling misunderstood or dismissed when discussing mental health concerns (Table 4, Question 6).

#### Communication with oncology teams

Patient comfort in discussing psychosocial needs with oncology providers varied. While some described becoming more open over time, others noted that these discussions rarely occurred unless initiated by the provider. The absence of proactive conversations about emotional well-being was viewed as a missed opportunity to normalize mental health care (Table 4, Question 7).

#### Strategies to reduce stigma and improve access

Patients proposed several solutions to reduce stigma and improve access. These included integrating mental health into routine cancer care, offering clinician training, launching public awareness efforts, and increasing institutional support for mental health services. Making services more affordable and expanding peer support networks were also key recommendations (Table 4, Questions 8 and 9).

#### Additional services and resources needed

Participants identified several unmet needs, such as greater availability of support groups, counselling services, and integrative therapies like mindfulness and art therapy. They also emphasized the importance of accessible, repeated, and culturally sensitive information delivery throughout the care journey (Table 4, Question 10).

## Discussion

This global survey of patients with cancer and oncology HCPs highlights widespread barriers to accessing and delivering PSO care across diverse settings. The main findings include: a) strong consensus among both patients and HCPs on the importance of PSO care, b) substantial gaps between perceived need and service delivery, c) structural barriers including inadequate staffing, limited training, and financial constraints, d) the pervasive influence of stigma and cultural norms on access and communication, and e) strikingly low prioritization and funding of PSO care within cancer research and institutional agendas. Collectively, these results identify the drivers of the relative neglect of humanistic cancer care as highlighted in the Commission on the Human Crisis of Cancer,^12^ and underscore the urgency of more fully integrating mental health into cancer care systems globally.

One of the most salient findings was the incongruity between perceived importance and actual delivery of PSO care. Notably, more than 80% of both patients (81.0%) and HCPs (85.2%) viewed psychosocial support as equally important as biomedical treatment, a level of endorsement that is strikingly high and warrants reflection. These figures may in part reflect self-selection bias, as individuals with strong opinions about or direct experience with psychosocial care may have been more likely to participate. However, the consistency of this finding across two independently recruited populations, patients and HCPs, surveyed through distinct channels, lends it credibility and suggests a genuine cross-stakeholder consensus. Furthermore, this convergence is itself a meaningful finding: it demonstrates that the barriers to PSO care identified in this study are not attributable to indifference or low perceived value, but rather to systemic and structural failures in translating acknowledged importance into routine practice. Against this backdrop, 63% of patients in our study reported not receiving any psychosocial support, and that more than half of HCPs reported PSO was not routinely provided at their institutions, underscores the magnitude of the implementation gap. Notably, our survey distinguished psychosocial care delivered within routine cancer care (e.g., communication, emotional support, and attention to distress by oncologists, nurses or social workers) from specialized mental health treatment. The findings therefore primarily reflect gaps in universal, humanistic care rather than access to specialist services. Ideally, this level of care should be consistently provided to all patients, yet our results suggest it is not systematically embedded in oncology practice. Prior literature has largely focused on utilization of specialized mental health services, which typically apply to a smaller subgroup of patients. Consistent with this, studies indicate that only a minority of patients ultimately receive psychotherapy or psychiatric care despite high distress levels.^14^^,^^15^ Rather than contradicting these findings, our results suggest that the care gap may arise earlier in the care pathway: insufficient routine psychosocial engagement within oncology teams may limit identification of distress and appropriate referral. This may be particularly pronounced in LMIC settings, where patients in our sample were significantly less likely to access mental health support.

A closer comparison of patient and provider perspectives across key domains reveals both meaningful convergences and instructive divergences. Both groups agreed strongly on the importance of PSO care, with over 80% of both patients and HCPs viewing psychosocial support as equally important as biomedical treatment, a convergence that underscores broad cross-stakeholder consensus on the value of PSO. Stigma was similarly recognized as a prevalent barrier by both groups, with over half of patients (57.0%) and HCPs (52.7%) reporting that mental health stigma was common in their settings. However, HCPs reported higher rates of cultural norms influencing mental health communication (74.3%) compared to patients (64.0%), suggesting that providers may perceive cultural barriers as more pervasive than patients themselves report, a divergence that may reflect providers’ broader systemic awareness or, alternatively, a tendency to attribute care gaps to cultural factors rather than structural ones. Conversely, while 63.5% of patients reported receiving no psychosocial support, only 53.2% of HCPs reported that PSO services were not routinely provided at their institutions, a gap that may indicate underestimation on the part of providers of how infrequently care reaches patients in practice. These group-level contrasts highlight the value of capturing both perspectives simultaneously and suggest that interventions targeting provider awareness of actual care receipt, alongside cultural competency training, may be particularly impactful. Future dyadic or institution-level studies, in which patients and their treating providers are surveyed in matched pairs, would allow more precise quantification of concordance and discordance and help identify where in the care pathway the most critical gaps arise.

Our study identified contributors to the PSO care gap that are consistent with previous publications, including patient-level financial barriers to accessing services^16^ and limited funding from governments and institutions to implement PSO.^17^^,^^18^ We also observed that patients who were unemployed or on disability were more likely to seek mental health care and to rate psychosocial support as highly important. Although causal relationships cannot be inferred, this pattern may indicate an association between psychosocial burden and impaired work participation in cancer survivorship. Improved integration of PSO care could therefore have broader economic relevance by supporting functional recovery and return-to-work, in addition to improving quality of life.

Another prominent barrier reported by both patients and HCPs was the limited PSO workforce and insufficient training for HCPs, with even greater deficits in LMICs.^17^, ^18^, ^19^ However, our results suggest that this gap is not primarily driven by lack of professional interest. Nearly half of HCPs in our sample (46.4%) perceived their colleagues to be motivated to pursue additional psychosocial oncology training, indicating that willingness to engage in PSO care is present. Rather, limited training opportunities and workforce shortages to reflect structural barriers, including insufficient institutional support, competing clinical demands, and the absence of standardized certification pathways.^20^ Our findings add to this literature and suggest that, without systems-level change, expansion of PSO services will remain constrained by human resource capacity and inadequate professional development.

Cultural and social factors further limit access to care. Over half of both patients and providers reported that stigma about mental health remained common in their settings, consistent with findings from systematic reviews and meta-analyses showing that public stigma towards mental health is widespread, with negative attitudes and exclusionary behaviors consistently documented across diverse populations and contexts.^21^^,^^22^ Patients described feeling judged, weak, or unsupported when seeking psychosocial care, and many described discomfort in discussing emotional needs with their oncology teams. Providers likewise acknowledged that stigma and cultural taboos shaped their own communication practices and patient receptivity. Notably, prior publications in oncology have largely focused on cancer-related stigma and its effects on accessing cancer care,^23^ or on mental-health stigma among people with pre-existing severe and persistent mental illness,^24^ but evidence regarding mental-health-related stigma associated with cancer-related emotional distress in the broader cancer population, and its impact on PSO access, remains limited. Patients with cancer and mental health needs face double stigma, when the human need for emotional support in the face of a medical crisis like cancer is self-evident.

Importantly, the survey identifies actionable points for intervention, including provider training to normalize mental health discussions and community campaigns to reduce stigma about cancer-related emotional distress. Interdisciplinary training would enable task-sharing as a key strategy to extend PSO coverage in resource-limited settings. There is a need not only for service expansion but also cultural shifts, both in public discourse and within clinical teams, to normalize emotional suffering and validate psychosocial care in cancer. This perspective aligns with the Lancet Commission on the Value of Death, which has argued for reframing dying, suffering, and grief as natural and legitimate aspects of the human experience that require collective acknowledgement and integration into health care and society.^25^ For this purpose, evidence-based, multicomponent interventions that address mental health stigma^26^ may be pivotal in increasing mental health literacy, fostering open communication within oncology care, and ultimately reducing barriers to PSO access.

However, our findings also draw attention to the limited funding of PSO research relative to overall cancer research. HCPs reported that less than 10% of national cancer research funding was allocated to psychosocial issues, and two-thirds viewed this investment as inadequate. This perception aligns with prior analyses indicating that only 2.14% of total funds were allocated to PSO research over 2016–2020.^27^ These findings highlight the potential for research advocacy and collaboration to strategically target funding gaps, including integration of PSO outcomes into biomedical studies.

The predictors of patient-reported barriers to accessing PSO care and HCP comfort in providing such care identified in our multivariable models was consistent with previous work showing lower PSO availability in LMICs,^19^ greater integration of psychosocial care within palliative care practice,^28^ and the association between increased professional involvement in PSO and stronger endorsement of the importance of addressing psychosocial needs.^29^ Overall, these findings suggest that interventions targeting provider engagement, interprofessional collaboration, and region-specific barriers may enhance PSO delivery globally.

Several limitations of this study should be acknowledged. First, the sample was not representative of all regions, particularly in the sample of patients, which included a disproportionately higher number of responses from North America, limiting generalizability. Importantly, this study was not designed to support country-level comparisons or to draw country-specific inferences; findings should be interpreted as exploratory and hypothesis-generating, reflecting the perspectives of a diverse but non-probability sample. The primary unit of analysis in our multivariable models is the individual respondent, and findings capture variation across income classifications, WHO regions, and health systems rather than between countries. Future studies with larger, regionally stratified samples are needed to enable robust cross-national comparisons. Second, the patient and HCP surveys were distributed through distinct channels without dyadic linkage between patient respondents and their treating HCP, which precluded individual-level concordance analyses between patient and provider perspectives. Third, the voluntary nature of participation raises the possibility of self-selection bias, where individuals with strong opinions, higher functional status or direct experience with psychosocial care were more likely to respond. Fourth, all findings are based on self-report, which may be influenced by recall bias, social desirability bias, or misinterpretation of survey questions. It should be noted, however, that patient-reported and provider-reported experience data are methodological standards in health services and psychosocial oncology research, valued precisely because they capture lived realities and implementation gaps that objective metrics often fail to reflect; in the context of barriers to care access, perception is itself the phenomenon of interest. Fifth, while the qualitative responses were rich, the brevity of some entries and use of automated translation may have resulted in a loss of nuance and contextual meaning. Finally, small sample sizes within some sociodemographic or regional subgroups led to wide confidence intervals in regression models, requiring cautious interpretation of subgroup-specific findings. Formal a priori power calculations are not standard practice for exploratory, cross-sectional surveys of this nature; however, we acknowledge that the sample size limits statistical precision, particularly in subgroup analyses, and reduces the likelihood of detecting small but potentially meaningful associations. Future studies should consider oversampling underrepresented regions and populations, prospective psychometric validation of these instruments, and incorporate longitudinal designs to assess changes over time.

Despite these limitations, this study makes a meaningful contribution to the global literature on cancer-related mental health. Comparative evidence on HCP gender differences and WHO-region variations in perceived psychosocial needs, cultural factors, and barriers is limited. Our results therefore contribute novel, global insights that warrants further investigation. It is also one of the first efforts to simultaneously capture patient and provider perspectives on psychosocial care across multiple world regions. By integrating patient and provider perspectives, our study also highlights the value of co-design approaches in planning PSO services and policy initiatives. It identifies that stigma, training gaps, and funding shortages are shared global challenges. We also explored perceived institutional prioritization of PSO and whether PSO is mandated within cancer services globally, contributing to a clearer picture of the magnitude of the Lancet Oncology Commission on the Human Crisis in Cancer from a global perspective.^12^ Our results support this call to reposition psychosocial care as a core component of cancer treatment and to develop context-specific strategies for implementation. This work lays a foundation for future advocacy, resource allocation, and policy development aimed at closing the global PSO care gap.

In conclusion, addressing the PSO care gap requires coordinated efforts across clinical practice, policy, training, and research. Our findings emphasize that improving access to psychosocial oncology care must go beyond individual provider capacity or patient willingness to seek help; it requires system-level investment, evidence-based psychosocial care guidelines, institutional commitment, integration of training programs, and cultural transformation. Global cancer strategies must recognize emotional well-being not as optional, but as essential to comprehensive cancer care.

## Contributors

Juan P. Borda: Conceptualization, Methodology, Writing—original draft, Validation, Visualization, Supervision, Project administration, Funding acquisition; Gilla K. Shapiro; Conceptualization, Methodology, Validation, Writing—review & editing, Funding acquisition; William Rosa: Conceptualization, Methodology, Validation, Writing—review & editing, Funding acquisition; Cristiane Bergerot: Conceptualization, Methodology, Validation, Writing—review & editing, Funding acquisition; Loreto del Pilar Fernandez: Conceptualization, Methodology, Validation, Writing—review & editing, Funding acquisition; Wendy WT Lam: Conceptualization, Methodology, Validation, Writing—review & editing, Funding acquisition; Anja Mehnert-Theuerkauf: Conceptualization, Methodology, Validation, Writing—review & editing, Funding acquisition; Jeffrey Dunn: Conceptualization, Methodology, Validation, Writing—review & editing, Funding acquisition; Hannah Lunnay: Conceptualization, Methodology, Validation, Writing—review & editing; Jean Jackson: Conceptualization, Methodology, Validation, Writing—review & editing; Julia Maues: Conceptualization, Methodology, Validation, Writing—review & editing; Surandran Veeraiah: Conceptualization, Methodology, Validation, Writing—review & editing; Paul Jacobse: Conceptualization, Methodology, Validation, Writing—review & editing; Chioma Azusu: Conceptualization, Methodology, Validation, Writing—review & editing, Funding acquisition; Cara MacInnis: Conceptualization, Methodology, Validation, Writing—review & editing, Madeline Li: Conceptualization, Methodology, Validation, Writing—review & editing, Supervision, Project Administration.

All authors had full access to all data in the study and verified the accuracy of the data reported. All authors read and approved the final version of the manuscript.

## Data sharing statement

Deidentified individual participant data collected for this study, along with a data dictionary defining each variable, will be made available to other researchers upon reasonable request to the corresponding author (juan.borda@lhsc.on.ca). Access will be granted to researchers who provide a methodologically sound proposal for analyses that are consistent with the objectives of the original study. Requests will be reviewed by the study investigators. Data will be shared without participant identifiers, following approval of a proposal and execution of a data access agreement.

## Declaration of interests

The authors declare that they have no known personal or financial conflicts of interest that could have appeared to influence the work reported in this paper.

## Appendix A Supplementary data

Apendix 1

Apendix 2

Apendix 3

Apendix 4
