# Supplementary material for: The global cancer mental health survey: insights from patient and provider experiences on psychosocial care access
Source: eClinicalMedicine. 2026 Jul 9;97:104047. doi: 10.1016/j.eclinm.2026.104047 (PMC13380114; doi:10.1016/j.eclinm.2026.104047)
Supplement: Apendix 1 [file mmc1.docx]

**Appendix 1. Patient Survey**

Dear Patient,

We are committed to understanding and addressing the challenges you might face in accessing psychosocial oncology services. Psychosocial oncology is a specialized field in cancer care that focuses on the emotional, social, and mental health needs of patients facing cancer. It addresses all aspects of emotional distress, from common feelings like sadness, fear, or spiritual concerns to more serious mental health issues, such as depression, anxiety, trauma, or psychosis.

Psychosocial oncology care may be provided through your cancer team, including your nurse and oncologist. Mental health care such as counseling may be provided by a range of professionals, including psychologists, social workers, psychiatrists, and counselors.

By completing this survey, you’ll help us improve the care we provide and make sure that we are addressing the needs of all patients and survivors. Your input is essential, and we deeply appreciate your time and honesty.

**Information About You and Your Cancer**

What is your age?

What is your gender?

- Male
- Female
- Non-binary/Third gender
- Prefer not to say
- Other (Specify)

In which country do you currently reside?

What is your ethnicity? (*Select all that apply*)

- Black or African Descent
- Asian
- Caucasian/White
- Hispanic, Latino, or Spanish Origin
- Middle Eastern or North African
- Native American or Indigenous
- Pacific Islander
- Other (Specify):

What is the highest level of education you have completed?

- No formal education
- Primary education
- Secondary education
- Vocational training
- Bachelor’s degree
- Master’s degree
- Doctoral degree
- Other (Specify)

What is your current employment status?

- Employed full-time
- Employed part-time
- Self-employed
- Unemployed
- Retired
- Student
- Homemaker
- On disability
- Other (Specify)

What is your current marital status?

- Single
- Married
- In a domestic partnership/civil union
- Divorced
- Widowed
- Other (Specify)

Do you have private health insurance?

- Yes
- No

Do you currently or have you ever had cancer?

- Yes
- No

What type of cancer were you diagnosed with?

*If you have had multiple diagnoses, please respond about your most recent diagnosis.*

When were you diagnosed?

*If you cannot remember the exact day and/or month, please give an approximate.*

*Enter date in MULTIVARIABLE/MM/DD format*

What is your current cancer treatment phase?

- Active treatment
- Remission
- Palliative

What is the current stage of your cancer?

- Early
- Advanced

**Mental Health Care**

Have you sought mental health support related to your cancer?

- Yes
- No

From which type of professionals?

- Psychologists
- Social workers
- Psychiatrists
- Counselors
- Other (Specify)

Have you faced any challenges in accessing mental health care from any of the following professionals?

- No, I have not faced any challenges
- Counselors
- Psychiatrists
- Psychologists
- Social workers
- Other (Specify)

Could you describe the specific challenges you have experienced? (e.g., scheduling difficulties, availability of services at different locations, transportation issues, etc.)

How would you rate the availability of mental health care services in your area?

- Very Good
- Good
- Fair
- Poor
- Very Poor

Do you feel that mental health care is given enough importance in your care setting?

- Yes
- Unsure
- No

Have you experienced any financial barriers to accessing such care?

- Yes
- No

Please describe any financial barriers you have experienced to accessing mental health care.

**Psychosocial Care**

Have you received psychosocial care from your cancer team?

- Yes
- No

Have you wanted to receive psychosocial care?

- Yes
- No

What psychosocial care would you have liked to receive?

How satisfied are you with the psychosocial care from your cancer team such as your oncologist or nurse?

- Very satisfied
- Somewhat satisfied
- Neutral / Not sure
- Somewhat unsatisfied
- Very unsatisfied

What parts of the psychosocial care you received were the most helpful?

What parts of the psychosocial care you received could have been improved?

Do you feel that psychosocial oncological care is given enough importance in your care setting?

- Yes
- Unsure
- No

**Culture and Stigma**

How important do you think psychosocial care is in cancer?

- Very important
- Somewhat important
- Neutral / Not sure
- Somewhat unimportant
- Very unimportant

How important do you think psychosocial care is compared to medical cancer treatment?

- Psychosocial care is much more important than medical care
- Psychosocial care is more important than medical care
- Psychosocial care is equally important as medical care
- Psychosocial care is less important than medical care
- Psychosocial care is much less important than medical care

How much do you think cultural values in your country affect healthcare providers’ willingness to talk about emotional or mental health issues?

- Strongly affect
- Somewhat affect
- Neutral
- Rarely affect
- Do not affect at all

How common is the stigma around emotional struggles and seeking mental health care for patients with cancer in your region?

- Very common
- Somewhat common
- Neutral
- Rare
- Very rare

What cultural challenges in your country make it harder to talk about and get support for emotional or mental health issues among cancer patients? (select all that apply)

- Stigma and taboos surrounding mental health and emotional suffering
- Cultural beliefs and practices
- Ideas about male or female roles
- Religious beliefs
- Language barriers
- Limited understanding and use of health information
- Lack of time for healthcare professionals
- None
- Other (Specify)

Have you ever felt stigma or judgment when seeking psychosocial or mental health care?

- Yes
- No

Please describe if you have ever felt stigma or judgment when seeking psychosocial or mental health care.

Do you feel comfortable discussing your psychosocial or mental health needs with your oncology care team?

- Yes
- No

Please explain your comfort level of discussing your psychosocial or mental health needs with your oncology care team.

In your opinion, can anything be done to reduce stigma around seeking psychosocial and mental health care?

- Yes
- No

Please explain what can be done to reduce stigma around seeking psychosocial and mental health care.

**Suggestions for Improvement**

What improvements would you suggest to make psychosocial and mental health care more accessible and acceptable for patients like you?

Are there any additional services or resources you believe should be offered to support psychosocial and mental health care for cancer patients?

Thank you for your participation. Your feedback will play a crucial role in enhancing our care services
