# Supplementary material for: The global cancer mental health survey: insights from patient and provider experiences on psychosocial care access
Source: eClinicalMedicine. 2026 Jul 9;97:104047. doi: 10.1016/j.eclinm.2026.104047 (PMC13380114; doi:10.1016/j.eclinm.2026.104047)
Supplement: Apendix 2 [file mmc2.docx]

**Appendix 2. Health Care Professional Survey**

Psychosocial oncology addresses emotional distress in cancer, defined as “a multifactorial unpleasant experience of a psychological (cognitive, behavioral, emotional), social, spiritual, and/or physical nature that may interfere with the ability to cope effectively with cancer, its physical symptoms, and its treatment.” This definition includes states of emotional distress that extend along a continuum, ranging from common feelings of spiritual and existential suffering, sadness, and fears, to more severe mental health problems such as depressive, anxiety, trauma and psychotic disorders. Psychosocial oncology care can be provided by oncologists and nurses, as well as mental health specialists such as psychologists, social workers, psychiatrists, and counselors.

**Section 1: Sociodemographic Information and Professional Background**

What is your gender?

- Male
- Female
- Non-binary/Third gender
- Prefer not to say
- Other (Specify)

What is your age? (Minimum: 0; Maximum: 130)

What is your ethnicity? (*Select all that apply*)

- Black or African Descent
- Asian
- Caucasian/White
- Hispanic, Latino, or Spanish Origin
- Middle Eastern or North African
- Native American or Indigenous
- Pacific Islander
- Other (Specify)

In which country do you primarily practice?

Do you work with or does your work impact cancer patients or survivors?

- Yes
- No

What is your primary professional role?

- Medical doctor
- Nurse or advanced practice nurse
- Psychiatrist
- Psychologist
- Social Worker
- Patient advocate
- Health care leader/Administrator
- Researcher
- Policymaker
- Chaplain/ Clergy/ Spiritual care
- Other

What is your specialization?

- Medical Oncology
- Radiation oncology
- Surgical oncology
- Psychosocial oncology
- Palliative care
- General medicine
- Other (specify)

How many years of experience do you have working with cancer patients?

What is your primary work setting?

- Public hospital or clinic
- Private hospital or clinic
- Hospice center
- Community health center
- Academic/Research institution
- Non-profit support organization
- Other (specify)

**Section 2: Clinical Practice**

How involved are you in providing psychosocial oncology care for your patients?

- Very involved
- Somewhat involved
- Not very involved
- Not involved at all

How much of your overall clinical time is spent providing psychosocial oncology care for your patients?

- Less than 25%
- 25-50%
- 51-75%
- More than 75%

Is mental health care routinely provided to all patients in your institution?

- Yes
- No

What proportion of your patients with cancer require mental health care?

- Less than 25%
- 25-50%
- 51-75%
- More than 75%

In your opinion, what proportion of your patients who have or had cancer are receiving sufficient mental health care?

- Less than 25%
- 25-50%
- 51-75%
- More than 75%

What proportion of family caregivers of your patients with cancer require mental health care?

- Less than 25%
- 25-50%
- 51-75%
- More than 75%

How do you perceive the importance of psychosocial oncology care relative to biomedical oncology care?

- Psychosocial care is much more important than medical care
- Psychosocial care is more important than medical care
- Psychosocial care is equally important as medical care
- Psychosocial care is less important than medical care
- Psychosocial care is much less important than medical care

Do you think there is sufficient prioritization for psychosocial oncology care in your institution?

- Yes
- Unsure
- No

What are the top 3 barriers to providing effective psychosocial oncology care in your practice or region (Please rank from 1-3)?

- Lack of funding
- Insufficient training
- Cultural stigma
- Limited access to services
- Low prioritization / attitude towards psychosocial care in healthcare
- Insufficient psychosocial oncology staff
- Lack of awareness about psychosocial oncology
- Lack of interest among physicians for referral
- Gaps in policy
- None
- Other (please specify)

**Section 3: Education and Training**

How comfortable are healthcare professionals in your institution in providing psychosocial care?

- Very comfortable
- Somewhat comfortable
- Neutral
- Somewhat uncomfortable
- Very uncomfortable

Have you received any training in psychosocial oncology?

- Yes, I have received training
- No, but I am interested
- No, not interested
- Not aware of such training

How would you rate the accessibility of training opportunities in psychosocial oncology for healthcare professionals in your country?

- Very accessible
- Somewhat accessible
- Neutral
- Somewhat inaccessible
- Very inaccessible

What are the 3 main factors limiting healthcare professionals’ access to such training opportunities in your country?

- Financial cost
- Geographical location
- Lack of awareness
- Lack of interest
- Institutional support
- Language barriers
- Unavailability of such training
- Workload and time constraints
- There are no factors limiting access
- Other: [Please specify]

How motivated are healthcare professionals in your institution to seek training in psychosocial oncology?

- Very motivated
- Somewhat motivated
- Neutral
- Somewhat unmotivated
- Very unmotivated

What is the main factor influencing whether psychosocial oncology training is prioritized at your institution?

- Institutional policies
- Costs
- Personal interest
- Lack of time
- Patient advocacy
- Other: [Please specify]

**Section 4: Health Systems**

What are the top 3 strategies to enhance psychosocial oncology care in your country? (Please rank 1-3)

- Training programs for oncologists and nurses
- Subsidize psychosocial oncology training programs
- Establish an emotional distress screening program
- Including a psychosocial oncology specialist in the oncology team
- Increasing psychosocial oncology specialist staffing
- Developing and implementing setting-specific guidelines
- Improving collaborative projects and services
- Enhancing community awareness and reducing stigma through educational campaigns
- Increase in funding for psychosocial oncology care
- Other (Please specify)

Are there any industry incentives for psychosocial oncology care in your institution?

- Many
- Some
- Few
- None
- Unsure

What mental health care disciplines do you have available at your institution (select all that apply)?

- Psychiatry
- Psychology
- Social work
- Mental health nursing
- Lay/peer support
- Chaplain / Clergy
- Other (please specify):

How sufficient is mental health care staffing at your institution?

- Very sufficient
- Sufficient
- Neutral
- Insufficient
- Very insufficient

What payment models for mental health care are currently in place for patients with cancer in your institution? (Check all that applies to you)

- Out-of-pocket payments
- Insurance-covered (With or without copayment)
- Government-funded
- Sliding scale fees
- Support from NGOs or projects
- Free services
- Not sure

**Section 5: Research**

What proportion of all oncology research funding do you think is allocated to psychosocial oncology care in your country?

- <1%
- 1-10%
- 10-20%
- >20%
- Don’t know

Is there sufficient funding for psychosocial oncology research in your country?

- Very sufficient
- Sufficient
- Neutral
- Insufficient
- Very insufficient

Do you assess psychosocial outcomes in your research?

- Yes
- No, but I plan to in the future
- No, psychosocial outcomes are not relevant in my research
- I do not conduct research

[If answer other than “I do not conduct research] In research conducted do you have collaborative partnerships with psychosocial oncology researchers?

- Yes
- No
- I do not conduct research

What do you think are the top 3 research priorities in psychosocial oncology?

- Psychosocial strategies in the prevention and detection of cancer
- Understanding the causes of psychosocial distress in patients with cancer
- Novel psychosocial interventions
- Impacts of psychosocial care on cancer outcomes
- Survivorship
- End-of-life care and bereavement
- Cross-cultural studies
- Something else, please specify

**Section 6: Culture**

How prevalent is the stigma related to emotional struggles and mental health care among patients with cancer in your country?

- Very high
- High
- Moderate
- Low
- Very low
- None

[If answer other than “none” selected above] What are the causes of stigma about emotional struggles and seeking mental health care patients with cancer in your country? (Select all that apply)

- Limited knowledge
- Cultural views
- Limited availability of mental health services
- Media influence
- Other:

To what extent do cultural values in your country influence healthcare providers’ willingness to engage in conversations about emotional or mental health issues with their patients?

- Strongly influence
- Somewhat influence
- Neutral / No influence
- Rarely influence
- Do not influence at all

What are the top 3 barriers that exist in your country that hinder effective communication about emotional or mental health issues for patients with cancer?

- Stigma and taboos surrounding mental health and emotional suffering
- Cultural beliefs and practices
- Ideas about male or female roles
- Religious beliefs
- Language barriers
- Limited understanding and use of health information
- Lack of time for healthcare professionals
- Other (describe)
- None

Thank you for your participation. Your feedback will play a crucial role in enhancing our care services.
