# Supplementary material for: The global cancer mental health survey: insights from patient and provider experiences on psychosocial care access
Source: eClinicalMedicine. 2026 Jul 9;97:104047. doi: 10.1016/j.eclinm.2026.104047 (PMC13380114; doi:10.1016/j.eclinm.2026.104047)
Supplement: Apendix 3 [file mmc3.docx]

**Appendix 3. Survey engagement statistics**

**HCP Survey**

- **Views**: 1978
- **Starts**: 589
- **Submissions**: 421
- **Complete surveys**: 237

**Patient Survey**

- **Views**: 1,260
- **Starts**: 514
- **Submissions**: 325
- **Complete surveys:** 200

**Views**: The total number of times the survey link was opened.

**Starts**: The number of respondents who moved past the Welcome screen.

**Submissions**: Submitted surveys, including cases where participants did not consent, were ineligible based on screening questions, or submitted after answering only the two mandatory questions leaving all others blank.

**Complete surveys**: Submitted responses where participants provided consent, met eligibility criteria, and included at least one non-blank (non-NA) answer. These were the responses in the final dataset used for analysis.
