# Supplementary material for: The global cancer mental health survey: insights from patient and provider experiences on psychosocial care access
Source: eClinicalMedicine. 2026 Jul 9;97:104047. doi: 10.1016/j.eclinm.2026.104047 (PMC13380114; doi:10.1016/j.eclinm.2026.104047)
Supplement: Apendix 4 [file mmc4.docx]

**Appendix 4. Table S4. Multivariable Associations Between Sociodemographic/Professional Predictors and HCPs Responses**

|  | **Prerceived need for mental health care - patients** | | **Sufficient mental health care provided to patients** | | **Prerceived need for mental health care - caregivers** | | **Perceived enough importance is given to PSO care** | | **Comfort level of HCPs in providing psychosocial care** | | **Perceived sufficiency of funding for PSO research** | | **Comfort discussing mental health needs** | | **Perceived stigma prevalence related to mental health in cancer** | | **Cultural values affect HCP willingness** | | **Trained in PSO** | |
| --- | --- | --- | --- | --- | --- | --- | --- | --- | --- | --- | --- | --- | --- | --- | --- | --- | --- | --- | --- | --- |
|  | OR | (95% CI) | OR | (95% CI) | OR | (95% CI) | OR | (95% CI) | OR | (95% CI) | OR | (95% CI) | OR | (95% CI) | OR | (95% CI) | OR | (95% CI) | OR | (95% CI) |
| **Male gender** | 0·48* | (0·23–0·99) | 0·83 | (0·38–1·81) | 0·49 | (0·23–1·03) | 1·08 | (0·44–2·64) | 1·90 | (0·83–4·51) | 1·29 | (0·51–3·40) | 1·90 | (0·83–4·51) | 1·34 | (0·60–3·09) | 0·99 | (0·48–2·05) | 1·44 | (0·53–4·07) |
| **Years of experience** | 1·01 | (0·98–1·04) | 0·98 | (0·94–1·01) | 0·99 | (0·95–1·02) | 0·96 | (0·93–1·00) | 1·05* | (1·01–1·09) | 1·01 | (0·98–1·06) | 1·05* | (1·01–1·09) | 0·97 | (0·94–1·01) | 1·01 | (0·98–1·05) | 1·08** | (1·03–1·13) |
| **Work in private setting** | 0·69 | (0·34–1·39) | 1·19 | (0·56–2·50) | 1·01 | (0·50–2·05) | 2·17 | (0·92–5·22) | 0·59 | (0·26–1·30) | 1·51 | (0·63–3·78) | 0·59 | (0·26–1·30) | 1·75 | (0·78–4·07) | 1·59 | (0·77–3·31) | 0·77 | (0·25–2·29) |
| **Work in research** | 0·59 | (0·22–1·57) | 1·36 | (0·52–3·55) | 0·41 | (0·14–1·11) | 1·59 | (0·50–5·06) | 0·69 | (0·23–2·19) | 1·39 | (0·43–5·41) | 0·69 | (0·23–2·18) | 0·68 | (0·25–1·93) | 0·59 | (0·23–1·55) | 0·73 | (0·18–2·89) |
| **Nurses** | 0·94 | (0·33–2·69) | 0·99 | (0·33–2·95) | 1·62 | (0·55–4·82) | 1·43 | (0·41–5·01) | 0·59 | (0·18–1·9) | 0·86 | (0·25–3·02) | 0·59 | (0·18–1·9) | 0·84 | (0·27–2·60) | 0·99 | (0·35–2·87) | 0·63 | (0·15–2·57) |
| **Other primary role** | 0·95 | (0·34–2·63) | 0·88 | (0·30–2·56) | 1·12 | (0·38–3·32) | 1·83 | (0·51–6·75) | 0·66 | (0·2–2·21) | 0·76 | (0·23–2·57) | 0·66 | (0·2–2·21) | 2·11 | (0·63–7·60) | 0·95 | (0·33–2·74) | 0·46 | (0·11–1·72) |
| **Psychiatrist / Psychologists** | 1·31 | (0·42–4·20) | 1·82 | (0·60–5·58) | 1·62 | (0·50–5·36) | 1·92 | (0·50–7·64) | 1·45 | (0·40–5·21) | 0·80 | (0·21–3·01) | 1·45 | (0·40–5·21) | 0·66 | (0·19–2·22) | 0·67 | (0·21–2·12) | 10·83* | (1·88–79·15) |
| **Social workers** | 1·45 | (0·31–7·01) | 1·10 | (0·24–4·96) | 1·37 | (0·31–6·12) | 1·43 | (0·23–8·62) | 0·33 | (0·07–1·64) | 0·40 | (0·07–2·20) | 0·33 | (0·07–1·64) | 0·55 | (0·11–2·63) | 0·22 | (0·04–1·10) | 3·71 | (0·41–43·84) |
| **Specialized in other fields of medicine** | 1·38 | (0·52–3·67) | 1·47 | (0·53–4·08) | 0·92 | (0·31–2·68) | 1·64 | (0·48–5·70) | 1·89 | (0·61–6·25) | 1·05 | (0·34–3·42) | 1·89 | (0·61–6·25) | 1·37 | (0·43–4·47) | 1·22 | (0·43–3·47) | 0·59 | (0·15–2·18) |
| **Specialized in palliative care** | 3·76* | (1·05–14·74) | 1·21 | (0·36–4·06) | 4·36* | (1·27–15·60) | 0·33 | (0·07–1·39) | 0·83 | (0·22–3·35) | 0·59 | (0·14–2·57) | 0·83 | (0·22–3·35) | 1·21 | (0·31–5·12) | 0·66 | (0·19–2·32) | 1·60 | (0·25–11·26) |
| **Non-specialized in PSO** | 0·59 | (0·18–1·96) | 0·42 | (0·12–1·41) | 0·72 | (0·21–2·40) | 0·70 | (0·17–2·86) | 0·65 | (0·17–2·44) | 1·49 | (0·38–6·06) | 0·65 | (0·17–2·44) | 0·97 | (0·27–3·53) | 0·76 | (0·22–2·62) | 2·53 | (0·50–13·83) |
| **LMIC** | 2·97* | (1·18–7·63) | 0·54 | (0·20–1·43) | 1·41 | (0·54–3·72) | 3·77* | (1·20–12·79) | 1·70 | (0·58–5·23) | 1·21 | (0·40–3·80) | 1·70 | (0·58–5·23) | 3·85* | (1·37–11·45) | 1·44 | (0·58–3·63) | 3·04 | (0·73–13·60) |
| **Africa** | 1·24 | (0·40–3·88) | 0·81 | (0·24–2·69) | 2·70 | (0·80–9·38) | 0·18* | (0·04–0·74) | 0·53 | (0·14–1·92) | 0·78 | (0·2–2·98) | 0·53 | (0·14–1·92) | 1·38 | (0·37–5·37) | 1·64 | (0·51–5·32) | 0·52 | (0·09–3·04) |
| **Easter Mediterranean** | 2·07 | (0·60–7·35) | 1·06 | (0·25–4·29) | 2·61 | (0·74–9·50) | 0·56 | (0·12–2·48) | 0·12* | (0·03–0·51) | 4·36 | (0·59–91·15) | 0·12 | (0·03–0·51)* | 1·05 | (0·25–4·73) | 0·25* | (0·07–0·94) | 0·12* | (0·01–0·80) |
| **Europe** | 0·63 | (0·22–1·84) | 1·42 | (0·48–4·20) | 0·40 | (0·14–1·17) | 3·83* | (1·13–13·71) | 2·16 | (0·65–7·77) | 1·37 | (0·39–5·17) | 2·16 | (0·65–7·77) | 1·78 | (0·59–5·47) | 0·87 | (0·31–2·47) | 3·24 | (0·81–13·71) |
| **South-East Asia** | 0·19 | (0·05–0·72) | 2·22 | (0·51–9·39) | 1·06 | (0·28–4·00) | 0·86 | (0·15–5·05) | 0·26 | (0·05–1·31) | 0·61 | (0·12–3·35) | 0·26 | (0·05–1·31) | 1·01 | (0·21–5·46) | 1·80 | (0·44–7·77) | 0·03 ** | (0·001–0·26) |
| **Western Pacific** | 1·48 | (0·62–3·57) | 0·89 | (0·32–2·09) | 0·72 | (0·29–1·74) | 4·33* | (1·46–13·95) | 0·89 | (0·34–2·40) | 0·73 | (0·26–2·07) | 0·89 | (0·34–2·40) | 1·41 | (0·55–3·73) | 0·67 | (0·29–1·55) | 0·88 | (0·24–3·23) |
| **Dedicated time for PSO 25-50%** | 1·25 | (0·61–2·57) | 1·32 | (0·61–2·91) | 0·82 | (0·39–1·71) | 1·41 | (0·60–3·35) | 1·72 | (0·75–4·02) | 1·25 | (0·52–3·06) | 1·72 | (0·750–4·02) | 1·45 | (0·65–3·26) | 1·48 | (0·71–3·09) | 2·81 | (1·01–8·24) |
| **Dedicated time for PSO 51-75%** | 2·80* | (1·11–7·17) | 1·95 | (0·76–5·01) | 1·56 | (0·63–3·90) | 1·34 | (0·47–3·86) | 1·66 | (0·63–4·51) | 2·01 | (0·63–7·12) | 1·66 | (0·63–4·51) | 1·41 | (0·52–3·91) | 4·45* | (1·72–11·80) | 1·46 | (0·37–5·91) |
| **Dedicated time for PSO more than 75%** | 3·25* | (1·27–8·52) | 4·68* | (1·77–12·76) | 2·12 | (0·84–5·49) | 3·32* | (1·07–11·08) | 4·39 | (1·57–13·09) | 0·91 | (0·31–2·69) | 4·39 | (1·57–13·09) | 2·26 | (0·79–6·75) | 3·99* | (1·53–10·70) | 2·65 | (0·62–11·42) |

Notes: OR = Odds ratio; CI = Confidence interval. Multivariable models adjusted for other listed covariates. Reference categories: female gender, public work setting, medical doctor role, oncology-related specialization, high-income country, WHO region = Americas, and <25% clinical time in psychosocial oncology; years of experience was modeled as a continuous variable. *p < 0·05; **p < 0·001
